# Reporter-Mediated Evaluation of the Circadian Oscillations of *SNAIL* Across In Vitro Models

**DOI:** 10.3390/clockssleep7040054

**Published:** 2025-09-28

**Authors:** Kaitlyn Chhe, Bhavna Kalyanaraman, Sophie A. Spielberger, Hui-Hsien Lin, Stephanie R. Taylor, Michelle E. Farkas

**Affiliations:** 1 Department of Chemistry, University of Massachusetts Amherst, Amherst, MA 01003, USA; 2 Department of Biology, University of Massachusetts Amherst, Amherst, MA 01003, USA; 3 Department of Computer Science, Colby College, Waterville, ME 04901, USA; srtaylor@colby.edu

**Keywords:** breast cancer, cancer biology, circadian rhythms, epithelial-to-mesenchymal transition (EMT), luciferase reporters, MCF7 cells, MCF10A cells, MDA-MB-231 cells, rhythmicity, SNAIL, U2OS cells

## Abstract

The protein SNAIL has been widely studied for its roles in promoting cancer invasion and resistance to apoptosis. There are multiple contributors to its expression, including self- and circadian regulation, and it has been posited that *SNAIL* oscillates in a circadian manner. Given the multiple factors involved, we sought to determine whether this is indeed the case. We developed a luciferase reporter that was used to demonstrate *SNAIL*’s rhythmic nature (*SNAIL:luc*) in the circadian model cell line, U2OS. Considering *SNAIL*’s relevance in breast cancer, we also assessed its oscillations in cellular models representing different levels of aggression. We incorporated the *SNAIL:luc* reporter in MCF10A breast epithelial cells, and MCF7 and MDA-MB-231 breast cancer cell lines, which are less and more aggressive, respectively. We found that *SNAIL* oscillations were present but weak in MCF7 and arrhythmic in MDA-MB-231 cells, correlating with those of core clock genes (*BMAL1* and *PER2*) in these models. Surprisingly, MCF10A cells, whose core clock genes possess robust circadian expression patterns, did not have rhythmic oscillations of *SNAIL*. Our findings suggest that *SNAIL* is under circadian control, but this is cell line/tissue dependent, setting the stage for additional studies to better understand the impacts of various factors contributing to its expression.

## 1. Introduction

Circadian rhythms are evolutionary adaptations that follow a roughly 24 h cycle. At the molecular level, circadian rhythms are governed by several clock components that form a complex autoregulatory feedback loop. The primary loop involves the proteins brain and muscle ARNT-like 1 (BMAL1) and circadian locomotor output cycles kaput (CLOCK), which serve as positive regulators. On the other hand, period 1/2/3 (PER1/2/3) and cryptochrome 1/2 (CRY1/2) proteins act as negative regulators of the molecular clock [1]. In the nucleus, BMAL1 and CLOCK heterodimerize and recognize E-box elements (5′-CANNTG-3′, where N = any nucleotide) in the promoters of clock-controlled genes (CCGs), including those of core circadian genes *PER* and *CRY*, to activate their transcription [1,2]. As PER and CRY proteins accumulate in the cytoplasm, they dimerize into PER:CRY complexes that translocate into the nucleus to impede CLOCK:BMAL1, resulting in the inhibition of their own expression [3].

In addition to their roles in maintaining cellular circadian rhythms, core clock genes (i.e., *BMAL1*, *CLOCK*, *PER*, and *CRY*) regulate CCGs and proteins responsible for several regulatory processes and key biochemical processes, such as DNA damage responses [4], cell organelle metabolism [5], and many others [6]. In many cases, this is the result of BMAL1:CLOCK binding to regulatory regions of CCGs. For example, the expression of c-MYC, a transcription factor that plays key roles in cell growth, proliferation, differentiation, and apoptosis [7], is controlled by BMAL1 and NPAS2 (a CLOCK paralog), which bind to the E-boxes present in the *c-MYC* promoter [8]. Meanwhile, CRY2 and FBXL3 cooperatively promote the ubiquitination and subsequent degradation of c-MYC [9]. BMAL1 and CLOCK also activate transcription of the *WEE1* gene, which encodes for WEE1, a G2/M inhibitor, by binding to E-boxes found in its promoter [10].

Disruption of circadian rhythms has been linked to various diseases, including cardiovascular [11], metabolic [12], and multiple cancer types [13,14,15,16]. In terms of cancer, multiple tumor types were found to have low levels of BMAL1, CLOCK, PER, and/or CRY [17,18,19,20,21]. At the same time, increasing the expression of these genes in tumor cells can enhance tumor sensitivity to therapeutics or have anti-tumorigenic effects [22,23,24,25,26]. Studies have also shown that circadian rhythms persist in some cancer types, and/or circadian proteins can aid in the proliferation of some tumors [27,28,29,30,31,32]. BMAL1 has been reported to aid the tumorigenesis and metastasis of cancer cells [28,29,33,34]. Overexpression of BMAL1 can activate the downstream Wnt/β-catenin signaling pathway, resulting in increased c-MYC expression [34]. This is one way of promoting epithelial-to-mesenchymal transition (EMT), resulting in cancer progression. EMT is the process by which epithelial cells acquire mesenchymal phenotypes (e.g., enhanced motility, reduced adherence) and also occurs normally during embryonic development and wound healing in adults [35]. On the other hand, a recent study reported that the BMAL1 gene can inactivate the TGF-β pathway by interacting with and inhibiting the translocation of SMAD, indirectly reducing the expression of SNAIL, and inactivating EMT [36].

The SNAIL family of zinc-finger transcription repressors includes SNAIL (SNAIL1), SLUG (SNAIL2), and SMUC (SNAIL3), which have been extensively studied for their roles in EMT, especially in breast cancer progression and reoccurrence [37]. NF-κB, SMAD3, and IKKα are some of the cell-signaling factors known to upregulate the expression of SNAIL [38]. In contrast, the tumor suppressor MTA3 has been shown to downregulate SNAIL expression [39,40]. Due to its role in promoting EMT, SNAIL serves as an important prognostic marker of malignancy in breast cancer. Its overexpression enables breast cells to become tumor-initiating cells and evade immune detection, and contributes to tumor aggression [37,41,42]. In addition to SNAIL being controlled by the factors described above, it has both direct and indirect interactions with circadian clock elements. The SNAIL-encoding *SNAI1* gene (also referred to as *SNAIL* and hereby referred to as such) contains two 5′-CACCTG-3′ E-box regions in its promoter sequence, which are key binding sites for the BMAL1:CLOCK heterodimer and support the possibility that the expression of *SNAIL* may be under circadian control [43]. Previous studies have shown that *BMAL1* expression is linked to that of *SNAIL*, specifically that its levels follow those of *BMAL1* in instances of both overexpression and knock-down [44,45]. There are two additional ways in which *SNAIL* expression is known to be regulated. The first is the ability of SNAIL to self-regulate its expression by binding to the E-box region of its own promoter [46]. The second is that *SNAIL* expression is also affected by differentiated embryo-chondrocyte 1 (DEC1), which has been recognized as an important negative regulator of the BMAL1:CLOCK heterodimer [47,48,49]. The involvement of multiple components in the regulation of *SNAIL* expression, including itself, and its association with the circadian core clock, makes it important to understand *SNAIL*’s expression patterns and investigate its circadian nature further.

Interestingly, studies have reported a circadian-like expression pattern of the SNAIL protein, which can be affected by perturbations of components involved in prominent cancer signaling pathways, such as MAPK, TGF-β, and Wnt pathways [50,51,52]. Western blotting and reverse transcription-quantitative polymerase chain reaction (RT-qPCR) experiments have been used to track the translation and transcription, respectively, of SNAIL across different cell lines including NIH-3T3 murine fibroblasts [43], human gingival fibroblasts (HGF-1) [43], BxPC-3 human pancreatic cancer cells [52], and MCF7 human breast cancer cells [50]. While these assays suggest that *SNAIL* expression is circadian, limitations in sampling frequency and experimental durations result in low-resolution and insufficient data, which makes it difficult to determine whether its nature is in fact rhythmic. Additionally, these assays do not provide enough information to assess circadian parameters such as the period and phase of the oscillations. Due to the interval length and dynamic nature of circadian rhythms, it is important to track transcriptional activity in a high-content manner over multiple cycles. These limitations can be addressed by using luciferase reporters, which have been widely used to track circadian processes. They have a large dynamic range and low background noise, enabling the detection of lower amplitude signals. Furthermore, luciferase reporters are adequately unstable so that reporters will not accumulate and misrepresent changes in gene expression [53]. The sensitivity and accuracy of luminescent reporters make them a reliable tool for studying biological rhythms at the cellular level.

We have previously reported that the strength of circadian rhythms may be inversely correlated to breast tumor aggressiveness (i.e., metastatic potential or risk) [54,55]. While these studies provided insights into the links between cancer severity and circadian rhythms, there is still a gap in understanding the circadian regulation of EMT factors, including SNAIL, and changes to their expression patterns with increasing malignancy. We sought to determine whether *SNAIL* oscillates rhythmically, and if so, whether its circadian nature is altered in different cancer models. For this purpose, we developed a luciferase reporter system to track human *SNAIL* promoter activity in a standard cell model used to study circadian rhythms (i.e., U2OS cells) [56,57,58,59,60] and across breast epithelial (i.e., MCF10A) and breast cancer cell lines of differing aggressiveness (i.e., MCF7 and MDA-MB-231) to better understand the connections between *SNAIL* oscillations and cancer. To obtain detailed information on the rhythmic nature of *SNAIL*, we performed our experimental analyses over extended time periods to better characterize the nature of the oscillations and obtain precise estimates of circadian parameters for *SNAIL*. We initially hypothesized that *SNAIL* transcription is circadian, and that more malignant breast cancer cell lines will have reduced or loss of *SNAIL* oscillations (i.e., MCF7 versus MDA-MB-231).

Since previous studies suggested the circadian nature of SNAIL, we first established that *SNAIL* expression oscillates in a model cell line used to study circadian rhythms (i.e., U2OS cells) and compared its oscillations to those of *Bmal1* and *Per2*. We report that *SNAIL* transcription oscillates in U2OS cells, and that while its period is similar to that of *Bmal1* and *Per2*, the phase of *SNAIL* is slightly advanced compared to that of *Per2* and delayed compared to *Bmal1*. We then explored the role of SNAIL as a potential mediator between circadian rhythms and tumor progression by comparing the oscillations of *SNAIL* across breast (including cancer) cell models (i.e., MCF10A, MCF7, and MDA-MB-231). We were surprised to find that *SNAIL* transcription does not oscillate in non-cancerous MCF10A cells, which possess oscillations of *Per2* and *Bmal1*. However, we found that *SNAIL* oscillates in the luminal A MCF7 cells, although there are data that are non-circadian, and does not in the triple-negative MDA-MB-231 breast cancer cell lines, largely correlating with our findings for the core clock genes. This study demonstrates the circadian nature of SNAIL and highlights the deviations that may occur, likely on account of the involvement of several other factors in its expression and regulation.

## 2. Results and Discussion

### 2.1. SNAIL Oscillations Can Be Tracked Using a Luciferase Reporter and Are Circadian in U2OS Cells

We first generated the *SNAIL* promoter reporter using standard molecular cloning methods. While our initial intent was to use a 769 base pair (bp) fragment from a plasmid containing the *SNAIL* sequence [39], following cloning into a pMA3160 lentiviral backbone containing luciferase [61], we found that we had obtained a truncated 340 bp sequence. The resulting construct, *SNAIL:luc*, was validated by restriction digest and whole plasmid sequencing. Comparison with the human SNAIL promoter revealed complete alignment between our truncated promoter plasmid and the endogenous sequence (Figure S1). Importantly, our promoter sequence contained the two 5′-CACGTG-3′ E-box regions present in the endogenous version.

We subsequently determined that the generated construct is sufficient for tracking *SNAIL* transcription and that *SNAIL* transcriptional activity oscillates using a standard in vitro model for circadian rhythms, U2OS (bone osteosarcoma) cells [56,57,58,59,60]. We stably transfected the reporter construct via lentiviral means, producing U2OS-*SNAIL:luc* cells. Following selection, the signal produced was validated via luciferase assay (Figure S2). Compared to the parental U2OS cells, U2OS-*SNAIL:luc* cells had a 22-fold higher level of bioluminescence intensity, which suggested the presence of luciferase in the reporter cell line.

**Figure 1:**
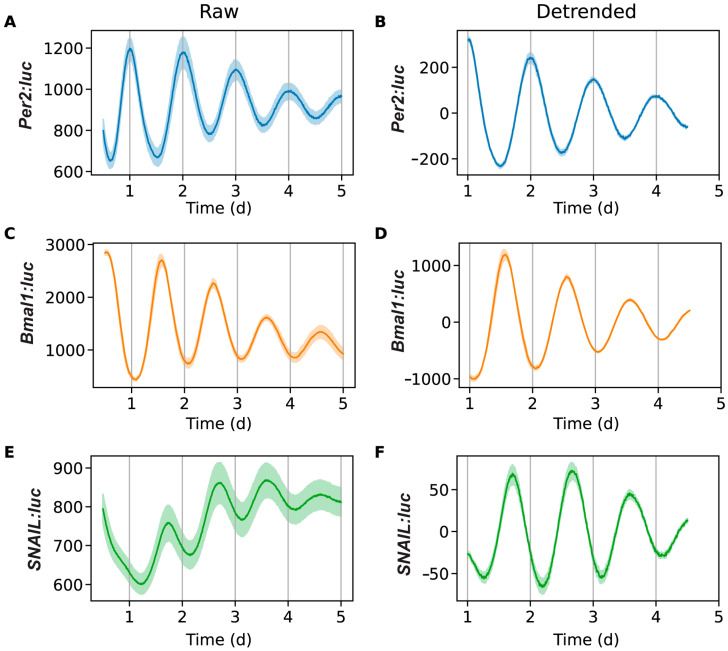
Bioluminescence time-series for *Per2:luc* (**A**,**B**), *Bmal1:luc* (**C**,**D**), and *SNAIL:luc* (**E**,**F**). Excluding a 12 h transient, shown are raw time-series (**A**,**C**,**E**) and time-series after de-trending (**B**,**D**,**F**) by removing the average of a 24 h moving window. The mean (raw or de-trended) time-series is plotted as a solid line, with the standard error of the mean as a semi-transparent envelope around it. (N = 3 for *Per2:luc*, N = 3 for *Bmal1:luc*, and N = 15 for *SNAIL:luc*).

**Figure 2:**
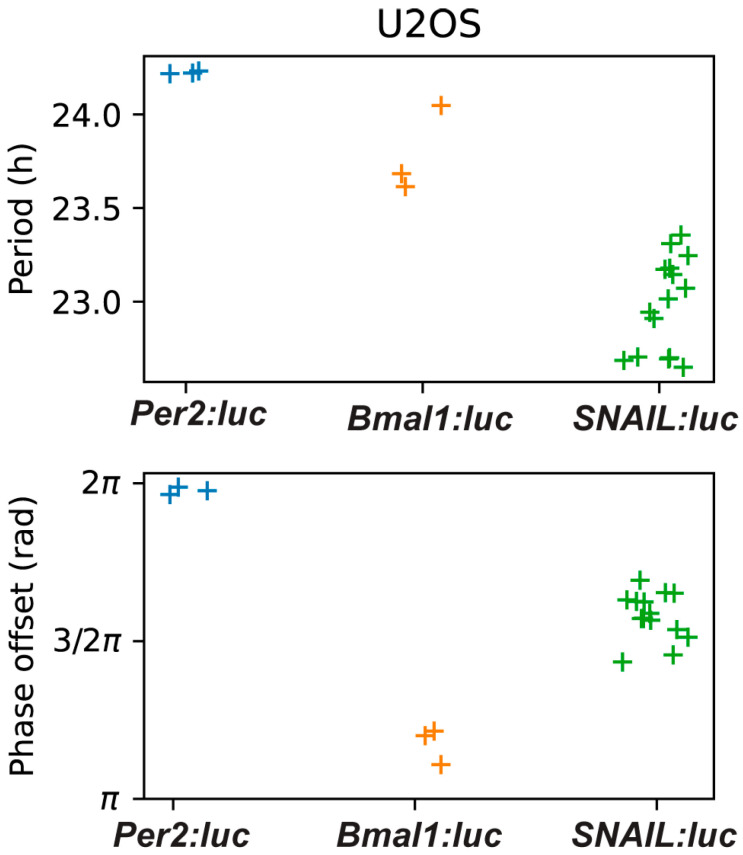
Shown are the period (**above**) and phase-offset (**below**) values estimated by fitting a damped cosine curve to de-trended *Per2:luc*, *Bmal1:luc*, and *SNAIL:luc* time-series. (N = 3 for *Per2:luc*, N = 3 for *Bmal1:luc*, and N = 15 for *SNAIL:luc*).

We used luminometry assays to track the oscillations of *SNAIL*-associated luciferase from our reporter. Preliminary experiments in U2OS-*SNAIL:luc* cells revealed that signals were both trackable and oscillatory. In subsequent experiments we performed parallel assessments, comparing *SNAIL* with *BMAL1* and *PER2*, using reporter cell lines previously generated by our lab (U2OS-*Bmal1:luc* and U2OS-*Per2:luc*, respectively) [62]. All three cell lines were synchronized via dexamethasone pulse for two hours, a common method used to synchronize U2OS cells [63,64,65,66,67]. Bioluminescence intensity was tracked for seven days. The signals were pre-processed by removing the first 24 h of raw data to remove the transient peak. Oscillations beyond five days were discarded, as they were generally determined to be too weak to analyze. Then, the data was de-trended by subtracting a 24 h window moving average (Figure 1 and Figure S3). From the de-trended data, circadian parameters were calculated, including the periods and phase offsets (Figure 2 and Figure S4). The period is the length of time between sequential peaks or troughs. The phase offset measures the time of the first peak relative to the dexamethasone pulse, which is the reference time (time = 0 h), so a phase of π indicates that the peak is one half of a cycle after the pulse (as is the case for *BMAL1*). A time-series was considered an outlier if the period or phase offset was greater than two standard deviations away from the mean for all recordings for a given reporter. Three U2OS-*SNAIL:luc* replicates (of 18) were deemed outliers.

We found the period of *SNAIL* to be slightly shorter than the typical circadian range of 23.5 to 24.5 h. The period was estimated to be 23.0 ± 0.24 h (mean ± standard deviation) by fitting a damped cosine curve to the de-trended time-series (Figure 2) and 23.1 ± 1.55 h when estimated by averaging the time differences between the first four peaks (Figure S4). The phase offset for *SNAIL*, found by fitting to a damped cosine curve, was 1.58 ± 0.07 π rad, indicating a reliable peak approximately three quarters of the way through each cycle. All recordings were rhythmic (*p* < 0.001 for all but one outlier recording, for which *p* < 0.003) according to an FFT-based test [68].

The bioluminescence intensities and the oscillations of *BMAL1* and *PER2* were consistent with previously reported results [60,62,63]. When *BMAL1* peaks, *PER2* troughs, and vice versa, because *PER2* represses *BMAL1* activity. Periods of *PER2* and *BMAL1* were determined to be 24.22 ± 0.01 h and 23.78 ± 0.19 h, respectively (each approximately one hour longer than that of *SNAIL*). These values align with period calculations for these genes in U2OS cells assessed previously [60,62,63,69,70]. Since *SNAIL* contains E-boxes in its promoter, we expected that its phase would be similar to that of *PER2*. However, we observed that the phase of SNAIL is advanced compared to that of *PER2* and delayed compared to that of *BMAL1*. The phase offset of *BMAL1* was 1.17 ± 0.04 π rad, with *SNAIL* following at 1.58 ± 0.07 π rad, and finally *PER2* at 1.98 ± 0.01 π rad (nearly at the beginning of the next cycle). Our results corroborate that *SNAIL* regulation is multifaceted, and that while the core circadian mechanism is a key component of *SNAIL* transcription, other factors may play roles in its timing, resulting in unanticipated offsets from core circadian components.

### 2.2. SNAIL Oscillations in Breast Epithelial and Breast Cancer Cell Lines Vary with Expression Levels and Aggressiveness

Since SNAIL is in part responsible for EMT and associated with cancer progression and metastasis, including in breast cancer, and breast cancer has many links to circadian rhythms and their alterations [71], we wished to assess its oscillations in breast (cancer) models. We opted to use MCF10A (a commonly used model for normal human mammary epithelial cells), MCF7 (luminal A cancer subtype, expressing estrogen and progesterone receptors (ER and PR, respectively), HER2-negative), and MDA-MB-231 (highly aggressive “triple-negative” claudin-low breast cancer subtype, lacking ER/PR/HER2) cells. Previous work from our group has shown an inverse correlation between *BMAL1* and *PER2* transcriptional activity and cancer aggressiveness [54,55,72]. We observed that these genes oscillate robustly in MCF10A (non-cancerous) cells, with dampened oscillations in MCF7 (slow-growing, less aggressive) cells, and no oscillations in the MDA-MB-231 (fast-growing and spreading, aggressive) cells. We transfected the *SNAIL* promoter reporter into these cell lines via lentiviral transfection. Luciferase assays were performed to validate the incorporation of the reporter in the cells (Figure S5). We found 13-, 25-, and 38-fold increases in the bioluminescence intensities of MCF10A-, MCF7-, and MDA-MB-231-*SNAIL:luc*, respectively, compared to the parental cell lines, indicating incorporation of luciferase.

**Figure 3:**
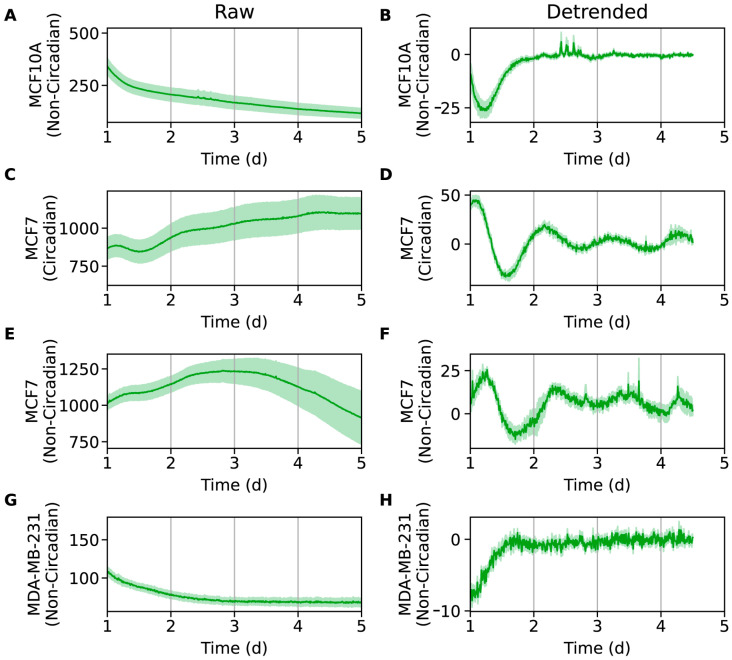
Bioluminescence time-series for *SNAIL:luc* in MCF10A (**A**,**B**), MCF7 (**C**–**F**), and MDA-MB-231 (**G**,**H**) cell lines. Excluding a 12 h transient, shown are raw time-series (**A**,**C**,**E**,**G**) and time-series after de-trending (**B**,**D**,**F**,**H**) by removing the average of a 24 h moving window. The mean (raw or de-trended) time-series is plotted as a solid line, with the standard error of the mean as a semi-transparent envelope around it. Seven MCF7 time-series were deemed circadian (**C**,**D**; rhythmic with a period in the range of 16 to 32 h). The remaining ten MCF7 (**E**,**F**) and all MCF10A (**A**,**B**) and MDA-MB-231 (**G**,**H**) time-series were non-circadian. (N = 15 for MCF10A-*SNAIL:luc*, N = 7 for MCF7-*SNAIL:luc* (circadian), N = 10 for MCF7-*SNAIL:luc* (non-circadian), N = 6 for MDA-MB-231-*SNAIL:luc*).

As with U2OS-*SNAIL:luc* cells, luminometry experiments were performed to evaluate oscillations in these cell lines. All three were synchronized via serum shock (cells starved for 14 h followed by a two-hour incubation in serum rich media), another common method used to synchronize cells [54,73,74]; alternative methods for synchronization were also used and are described further below. The data was processed as described for U2OS-*SNAIL:luc* to generate de-trended oscillations (Figure 3 and Figure S6). Despite finding that MCF10A cells possess rhythmic oscillations for *Bmal1:luc* and *Per2:luc* previously [72], we surprisingly did not observe oscillations for *SNAIL:luc*. We also employed other established synchronization methods, specifically dexamethasone [63,64,65,66,67] and forskolin pulses [75], which also did not result in any rhythmic signals (Figure S7A–D). Although SNAIL is expressed in MCF10A cells, its expression is quite low, and less than in MCF7 and MDA-MB-231 cell lines [76]. It is plausible that while *SNAIL*’s transcriptional activity oscillates in MCF10A cells, its amplitudes are too low to detect. This hypothesis can be tested by overexpressing *SNAIL* in MCF10A cells in the future, which may result in traceable oscillations.

Luminometry data from MCF7-*SNAIL:luc* cells yielded data whose de-trended signals were qualitatively similar, but with rhythms more prominent in some than others. We sought to identify signals that were low-amplitude circadian oscillations, so we chose criteria that were somewhat lenient. For each replicate, we computed the degree of rhythmicity using an FFT-based test that quantifies the relative strength of the circadian frequency [68]. Rhythmic replicates (*p* < 0.1) that fit a damped cosine curve well (coefficient of determination > 0.7) with a period in the range of 16 to 32 h were classified as being circadian (Figure 3D and Figure S6D). All others were non-circadian (Figure 3F and Figure S6F). Seven MCF7-*SNAIL:luc* replicates showed weak circadian oscillations, and ten were deemed non-circadian. Although oscillations are clearer in the recordings deemed circadian, there is a striking visual similarity across all recordings. Between this similarity and the observation that the non-circadian replicates were lower amplitude, it is plausible that all replicates were circadian, but that some had signals too weak to be clearly analyzed and deemed circadian. In prior work, we showed that both *Bmal1:luc* and *Per2:luc* signals in MCF7 cells displayed damped but rhythmic oscillations [54,77]. Our findings for *SNAIL:luc* align with those results. Synchronization by dexamethasone pulse did not result in detectable oscillations (Figure S7E,F).

MDA-MB-231 cells did not show any *SNAIL* oscillations following synchronization by serum shock (Figure 3G,H and Figure S6G,H) or dexamethasone pulse (Figure S7G,H). Due to the lack of oscillations, we did not perform period or phase calculations on these data. While MDA-MB-231 cells express higher levels of SNAIL [78,79,80], previous work showed that core circadian gene expression for *Bmal1* and *Per2* do not oscillate in them [54,77]. Hence, because the core clock mechanism may no longer be functioning normally in these cells, it is not surprising that *SNAIL* expression is driven only by non-circadian factors here.

## 3. Materials and Methods

### 3.1. Plasmid Construction

To generate a lentiviral plasmid expressing *SNAIL:luciferase* (*SNAIL:luc*), a 769 bp human *SNAIL* promoter fragment was PCR-amplified from a pGL2-Basic plasmid obtained from Addgene (Plasmid #31694, deposited by Dr. Naoyuki Fujita) [39]. The primer sequences used to amplify the *SNAIL* fragment were: Forward primer (containing EcoRI restriction site, underlined) = 5′-CCG GAA TTC AGG TGA CCC GCC TCT TAA C-3′ and reverse primer (containing NotI restriction site, underlined) = 5′-ATA AGA ATG CGG CCG CGG GCG GGG CCT TAT C-3′. The *SNAIL* promoter fragment was then purified and subcloned into a pMA3160 lentiviral construct (Addgene plasmid #35043, deposited by Dr. Mikhail Alexeyev) [61] using the restriction sites EcoRI and NotI, located upstream of the luciferase sequence. The recombinant *SNAIL:luc* plasmid was transformed into an electrocompetent Stbl3 strain of *E.coli* and then extracted by a GeneJET plasmid miniprep kit (Thermo Fisher Scientific, Waltham, MA, USA; #K0501). The recombinant plasmid was validated using restriction digestion with EcoRI/NotI and further validated using Sanger and whole plasmid sequencing (Azenta Life Sciences, Waltham, MA, USA).

### 3.2. Cell Culture

U2OS cells (ATCC) were cultured in DMEM (Gibco, Waltham, MA, USA) supplemented with 10% fetal bovine serum (FBS; Corning, Corning, NY, USA), 100 U/mL penicillin-streptomycin (Gibco), 2 mM L-glutamine (Gibco), 1 mM sodium pyruvate (Gibco), and 1X non-essential amino acids (Cytiva, Marlborough, MA, USA). MCF7 and MDA-MB-231 cells (ATCC) were cultured in DMEM supplemented with 10% FBS, 100 U/mL penicillin-streptomycin, and 2 mM L-glutamine. MCF10A cells were obtained from the Barbara Ann Karmanos Cancer Institute (Detroit, MI, USA). MCF10A cells were cultured in DMEM/F12 (Gibco) supplemented with 5% FBS, 100 U/mL penicillin-streptomycin, 2 mM L-glutamine, 15 µg/mL gentamycin (Fisher Bioreagents, Waltham, MA, USA), 10 µg/mL insulin (MP Biomedicals, Irvine, CA, USA), 20 ng/mL human epidermal growth factor (EGF; Gibco), 0.1 µg/mL cholera enterotoxin (Sigma-Aldrich, St. Louis, MO, USA), and 0.5 µg/mL hydrocortisone (Sigma-Aldrich). HEK293T cells were cultured in DMEM/F12 supplemented with 10% FBS and 100 U/mL penicillin-streptomycin. All cell lines were cultured at 37 °C in a 5% CO_2_ atmosphere.

### 3.3. Lentiviral Transduction

Lentiviral transductions for U2OS and MDA-MB-231 cells were performed following protocols established previously [60]. HEK293T cells were plated in 60 mm dishes at a density of 2.5 × 10^6^ cells per 60 mm dish. At 60–70% confluence the cells were transiently transfected with the *SNAIL:luc* transfer plasmid (3 µg per dish), pMD2.G envelope plasmid (2 µg per dish), and psPAX2 packaging plasmid (and 3 µg per dish) with Lipofectamine 3000 (Invitrogen) following the manufacturer’s protocol. The pMD2.G (Addgene plasmid #12259; http://n2t.net/addgene:12259; accessed on 20 January 2025; RRID:Addgene_12259) and psPAX2 (Addgene plasmid #12260; http://n2t.net/addgene:12260; accessed on 20 January 2025; RRID:Addgene_12260) plasmids were gifts from Didier Trono. Target cells were plated in T25 flasks at a density of 6 × 10^5^ cells/flask and transfected at 70–80% confluence.

For the infection, viral supernatant was collected, filtered through a 0.45 μm filter, and combined with fresh target cell media in a 1:1 ratio along with polybrene (4 µg/mL; Sigma-Aldrich). The culture media was removed, and 6 mL of viral media was added to each flask. Infections were repeated every 12 h for 2 days for a total of four infections. Cells were treated with media containing 4 µg/mL puromycin (Gibco) 48 h after the last infection. Cells were selected over 4–6 weeks, replacing the media with fresh puromycin-containing media twice a week.

MCF7 and MCF10A cells were transduced with a virus expressing the *SNAIL:luc* fragment, generated and obtained from VectorBuilder (Chicago, IL, USA). First, we optimized the multiplicity of infection (MOI) using a lentivirus that induces green fluorescent protein (GFP) and mCherry expression. MCF7 and MCF10A cells were plated in 24-well plates at a density of 5 × 10^4^ cells per well. The cells were infected with the lentivirus at MOIs of 5, 10, and 15 for MCF10A, and 1, 2, and 5 for MCF7 cells. Negative controls were not infected with the virus. We then compared the GFP and mCherry levels of the infected cells compared to the negative controls. We selected the MOIs that resulted in the highest GFP and mCherry fluorescence intensities with minimal cell death to infect cells with the *SNAIL:luc* lentiviruses.

Target cells were plated at a density of 6 × 10^5^ cells per flask. When 70–80% confluent, both cell lines were infected at an MOI of 5. The virus was diluted in target media with polybrene (8 µg/mL for MCF7 cells and 10 µg/mL for MCF10A cells). The cells were selected with target culture media containing puromycin (1 µg/mL for MCF7 cells and 4 µg/mL for MCF10A cells) 48 h after infection. Selection was carried out for 4–6 weeks, after which cells were expanded, and validated via luciferase assay.

### 3.4. Cell Synchronization and Bioluminescence Recording

Cells were plated in 35 mm dishes at a density of 2 × 10^5^ cells/mL and were synchronized when they were confluent. U2OS-derived cells (-*Bmal1:luc*, -*Per2:luc*, -*SNAIL:luc*) were synchronized with 100 nM dexamethasone (Sigma-Aldrich) dissolved in U2OS media for two hours. MCF10A-, MCF7, and MDA-MB-231-*SNAIL:luc* were synchronized via serum shock. First, the cells were starved in DMEM (for MCF7 and MDA-MB-231) or DMEM/F12 (for MCF10A) medium without any growth supplements for 14 h. Then the starvation media was replaced with growth media containing 50% FBS for 2 h. To test alternative synchronization methods, MCF10A, MCF7, and MDA-MB-231 cells were starved for 14 h in their respective media and synchronized with 100 nM dexamethasone or 10 μM forskolin (MCF10A only; Thermo Scientific, Waltham, MA, USA) for two hours.

After synchronization, the dexamethasone- or forskolin-containing or serum-rich media was replaced with recording media. Recording media for U2OS-derived cell lines was made with 11.25 mg/mL powdered DMEM (Sigma-Aldrich), 4 mM sodium bicarbonate (Fisher Bioreagents), 5% FBS, 10 mM HEPES (Cytiva), 50 U/mL penicillin-streptomycin, and 0.5 mM D-luciferin (Thermo Scientific) dissolved in autoclaved Millipore water. To make MCF7- and MDA-MB-231-*SNAIL:luc* recording media, 13.5 mg/mL of powdered DMEM, 1 mM sodium pyruvate (Gibco), 5% FBS, 10 mM HEPES, 100 U/mL penicillin-streptomycin, and 0.5 mM D-luciferin were dissolved in autoclaved Millipore water. MCF10A cells were recorded in phenol-red free DMEM/F12 containing 20% of normal growth supplement concentrations (0.4 mM L-glutamine, 3 mg/mL gentamycin, 2 mg/mL insulin, 4 ng/mL human EGF, 20 ng/mL cholera enterotoxin, 100 ng/mL hydrocortisone and 1% FBS), in addition to 6.5 mM sodium bicarbonate, 10 mM HEPES, 50 U/mL penicillin-streptomycin, and 0.5 mM D-luciferin. All recording medias were filtered through a 0.2 µm filter before adding to the cells. The dishes were sealed with 40 mm cover glasses and autoclaved silicone vacuum grease and monitored for 7 days using a LumiCycle 32 (Actimetrics, Wilmette, IL, USA) at 36.5 °C.

### 3.5. Data Analysis

Each bioluminescence time-series was processed by removing a 12 h transient from the beginning and by discarding oscillations after 5 days (deemed generally too weak to analyze), de-trended by removing the average of a 24 h moving window, and then fit to a damped cosine curve with a linear baseline, $Ae^{-\lambda t}\cos\left(\frac{2\pi }{\tau }-\theta \right)+c_{0}+c_{1}t$. The coefficient of determination was used to quantify the goodness of fit. Before reporting the period τ, phase θ, and average time-series for recordings from U2OS cells, three time-series using the SNAIL reporter were excluded as outliers. A time-series was considered an outlier if the goodness of fit was less than 0.85 or its estimated period or phase were more than two standard deviations of the mean across all replicates for the given reporter, cell line, and method of synchronization.

## 4. Conclusions

The *SNAIL* gene serves as an important prognostic marker for breast cancer malignancy, due to its role in promoting EMT. Evidence has suggested that *SNAIL* may also be under circadian control, on account of BMAL1:CLOCK binding sites in its promoter, and indirect interactions with core clock components. The other elements contributing to *SNAIL*’s expression patterns, including its self-regulation, and the roles of signaling factors, highlight its complexity. Previous work used Western blotting and RT-PCR to imply SNAIL’s circadian nature at the translational and transcriptional levels. However, due to limited experimental duration and infrequency of sampling, rhythmicity and circadian parameters (e.g., period and phase) cannot be determined. Therefore, to track and determine the characteristics of its oscillations, we used luciferase reporters, which enabled data collection in a high-content manner over multiple cycles.

In our work, we established a *SNAIL* promoter reporter construct (*SNAIL:luc*) that was used to determine that *SNAIL* oscillates in a circadian manner using U2OS cells, a well-established model for studying circadian rhythms. We found that *SNAIL* had a period of approximately 23 h, and exhibited a phase advance compared to *PER2*, and a phase delay compared to *BMAL1*. While this study focused on transcriptional regulation, the circadian expression of SNAIL at the translational level could also be modulated by the core clock. Further investigation into the mechanisms that control SNAIL protein translation efficiency and stability could provide additional insights into the links between SNAIL and circadian rhythms. Considering SNAIL’s role and the clock’s changes in breast cancer malignancy, we also evaluated the oscillations of *SNAIL* across breast non-cancerous and cancer cells with different levels of aggressiveness. We observed that MCF7 cells exhibited weak circadian oscillations for *SNAIL*, while *SNAIL* expression in MDA-MB-231 cells was arrhythmic, both largely corresponding with prior findings for core clock genes *BMAL1* and *PER2*. Interestingly, while the clock is functional in MCF10A cells, we did not observe rhythmic signals for *SNAIL*, possibly due to the very low levels present and its amplitudes being below the detection limit.

Taken together, our results suggest that *SNAIL* transcription is partially under circadian regulation and that additional regulatory mechanisms may contribute to its deviations from expected timing based on the core clock. Further, when the core clock itself is disrupted or oscillations are absent (i.e., in MDA-MB-231 cells), the other factors likely take control of SNAIL expression and presence. Our findings highlight the need to utilize advanced methodologies, such as luciferase reporters, to provide insights into the complex interplay between circadian rhythms and cancer progression. Using reporters, we accurately estimated the period of *SNAIL* and assessed its phase relationships, important characteristics that provide crucial information on the circadian control of SNAIL. Further studies should be performed to understand the connection between the clock and SNAIL, and in the context of breast cancer, whether the core clock and/or other factors control SNAIL expression and to what extent. Nonetheless, the presence of circadian oscillations in MCF7 cells suggests that our reporter strategy could be used as a screening tool to monitor the progression of early-stage cancers and be used in conjunction with other assays to probe clock-controlled disease mechanisms in patient-derived cell culture models. The tracking of circadian rhythms and cancer-associated genes in these models can help elucidate the role(s) of this dynamic process in cancer progression, which in turn can lead to novel therapeutic strategies targeting circadian-driven components or the modification of existing ones to account for timing in cancer treatments, thereby improving patient prognoses.

## Data Availability

The original data presented in the study are openly available in ScholarWorks at https://hdl.handle.net/20.500.14394/56741 (accessed on 26 June 2025).

## Supplementary Materials

The following supporting information can be downloaded at https://www.mdpi.com/article/10.3390/clockssleep7040054/s1.

## Abbreviation

The following abbreviations are used in this manuscript:

BMAL1	Brain and muscle Arnt-like 1
CLOCK	Circadian locomotor output cycles kaput
PER	Period
CRY	Cryptochrome
EMT	Epithelial to mesenchymal transition
DEC1	Differentiated embryo-chondrocyte 1
RT-qPCR	Reverse transcription-quantitative polymerase chain reaction
HGF-1	Human gingival fibroblasts
luc	Luciferase
FBS	Fetal bovine serum
MOI	Multiplicity of infection
