# Supplementary material for: Reporter-Mediated Evaluation of the Circadian Oscillations of SNAIL Across In Vitro Models
Source: Clocks Sleep. 2025 Sep 28;7(4):54. doi: 10.3390/clockssleep7040054 (PMC12550899; doi:10.3390/clockssleep7040054)
Supplement: Supplementary file 1 [file clockssleep-07-00054-s001.zip › clockssleep-3755028-supplementary.pdf]

Supplementary Materials

Reporter-Mediated Evaluation of the Circadian Oscillations of *SNAIL* Across *In Vitro* Models

Luciferase assay

Following lentiviral transduction and puromycin selection, *SNAIL:luc* reporter cell lines were validated with ONE-Glo EX Luciferase Assay System (Promega) following the manufacturer's protocol. Briefly, cells were plated in a 96-well plate at a density of 1 x 10^5 cells per well. When the cells were confluent (approximately 24 hours after plating), they were washed with phosphate buffered saline (PBS; Gibco), then fresh cell culture media and ONE-Glo EX Reagent were added to each well in a 1:1 ratio (100 µL each). The cells were incubated at ambient temperature for 3 minutes and bioluminescence intensity was measured with a SpectraMax iD3 plate reader. The average reading across three biological replicates was calculated and normalized to the respective non-transfected controls for each cell line to determine relative bioluminescence intensity.

```
Human SNAIL promoter        1 T---CAGGTGACCCGCCTCTTAACGGTCGCCGCGTCCCGTCTCTCCCCCACAAAAGCACA
SNAIL_luciferase pMA3160    1 AATTCAGGTGACCCGCCTCTTAACGGTCGCCGCGTCCCGTCTCTCCCCCACCAAAGCACA

Human SNAIL promoter       58 CTTCCCTTTGCATTGTAATTATCTGTTTACTTCGTCTGTCTCCCTCACTGGACCAGAAGC
SNAIL_luciferase pMA3160   61 CTTCCCTTTGCATTGTAATTATCTGTTTACTTCGTCTGTCTCCCTCACTGGACCAGAAGC

Human SNAIL promoter      118 TACCCTTCGGGAGAGGCTCTGAGTGTTCTGTCCGGGGCTGTGCCCTGGCCCCAAGTACAG
SNAIL_luciferase pMA3160  121 TACCCTTCGGGAGAGGCTCTGAGTGTTCTGTCCGGGGCTGTGCCCTGGCCCCAGGTACAG

Human SNAIL promoter      178 TGCCCCCACACGTGCTGGGCGCTCCGTAAACACTGGATAAGGGAAGGAACGGGTGCTCTT
SNAIL_luciferase pMA3160  181 TGCC-CCACACGTGCTGGGCGCTCCGTAAACACTGGATAAGGGAAGGAACGGGTGCTCTT

Human SNAIL promoter      238 GGCTAGCTGGGCCAGGCTGCTTTGCAAAAAGGCCGTGGCATTTCAAGCCGCCGAGAGCCA
SNAIL_luciferase pMA3160  240 GGCTAGCTGGGCCAGGCTGCTTTGCAAAAAGGCCGTGGCATTTCAAGTCGCCGAGAGCCA

Human SNAIL promoter      298 CGTGCGGTGTCCCTTTCCTCGCTTCCTCCCCAGTGATGTGCGTTTCCCTCGTCAATGCCA
SNAIL_luciferase pMA3160  300 CGTGCGGTGTCCCTTTCCTCGCTTCCTCCCCAGCGATGTGCGTTTCCCTCGTCAATGCCA

Human SNAIL promoter      358 CGCTC-TCCAGGCGCCAGCCGGGCGGAGGAAATCTCCGCCCCCTCCCAAGCCCGAGGCGG
SNAIL_luciferase pMA3160  360 AGCTTCTCGAGG---------GGCGG-GGCCTTATCTGCCCC-------GCCC-------

Human SNAIL promoter      417 GGGCGGGCGTCGGAAGGTCAGGTG
SNAIL_luciferase pMA3160  396 ----------------------GC
```

Figure S1. Sequencing results comparing the *SNAIL* promoter gene in the recombinant plasmid (SNAIL_1 luciferase pMA3160) to the human *SNAIL* promoter. Alignments between the two are highlighted in black.

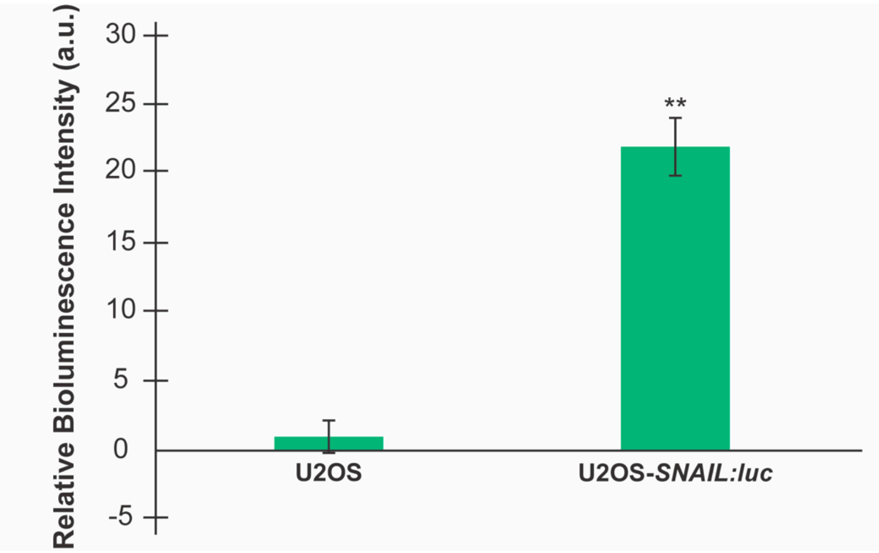

Figure S2. U2OS-*SNAIL:luc* validation. Non-transfected and reporter U2OS cell lines were assessed via a luciferase assay. Data shown for each condition is an average of three biological replicates (N=3) plotted with the standard error of the mean. A t-test was performed to compare the averages of the biological replicates for each cell line (NS indicates "not significant", ** $p <$ 0.01).

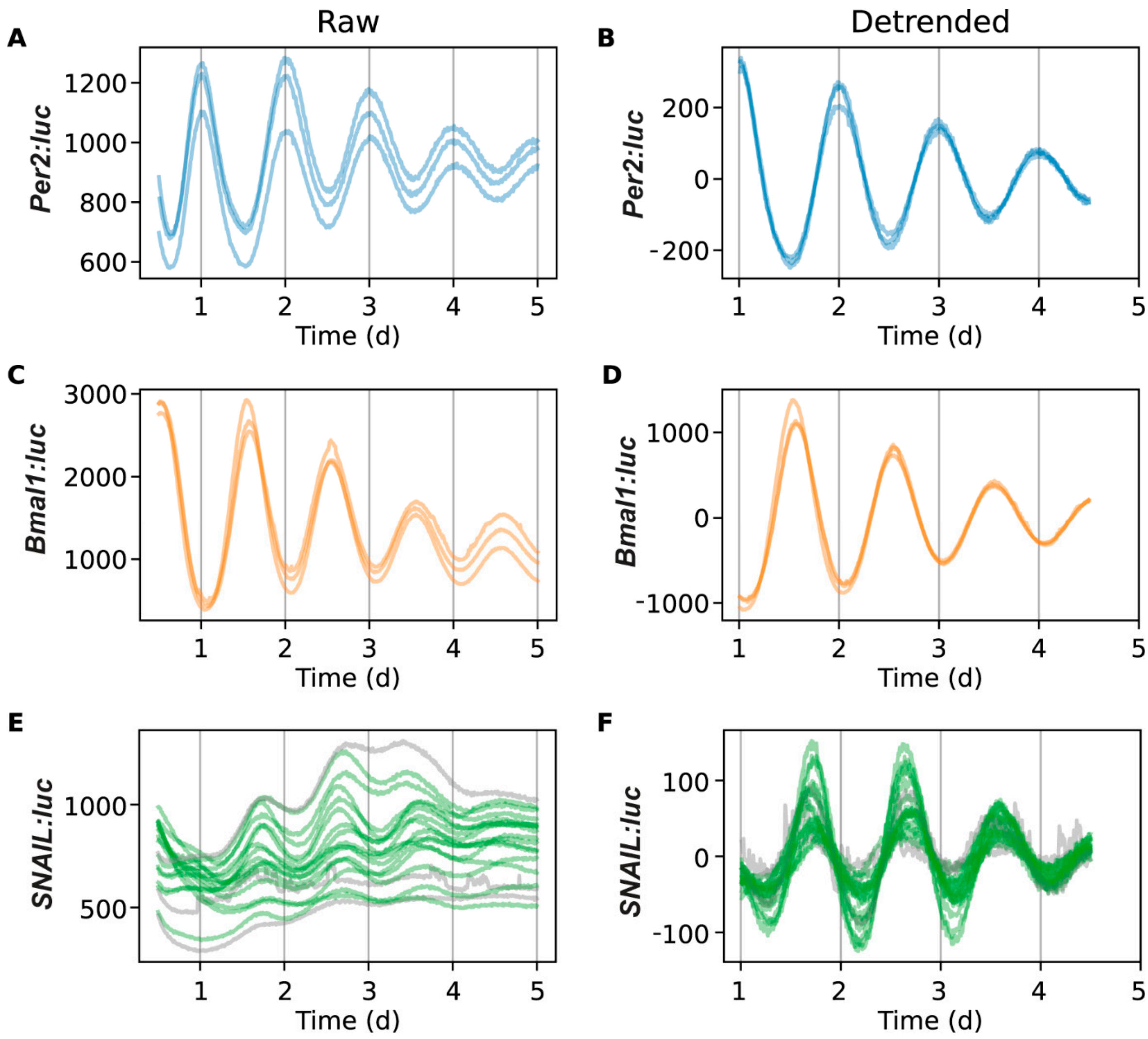

Figure S3. Individual bioluminescence time-series for *Per2:luc* (A,B), *Bmal1:luc* (C,D), and *SNAIL:luc* (E,F). Shown are the raw time-series excluding a 12-h transient, (A,C,E) and time-series after de-trending by removing the average of a 24-h moving window (B,D,F). (N=3 for *Per2:luc*, N=3 for *Bmal1:luc*, and N=18 for *SNAIL:luc*, for which three are outliers and are shown in gray).

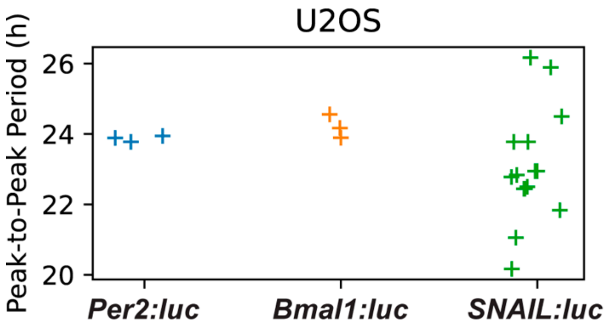

Figure S4. Shown are the periods of *Per2:luc*, *Bmal1:luc*, and *SNAIL:luc* time-series as estimated by the average difference in timing of the first four peaks starting 24 hours after the recording began.

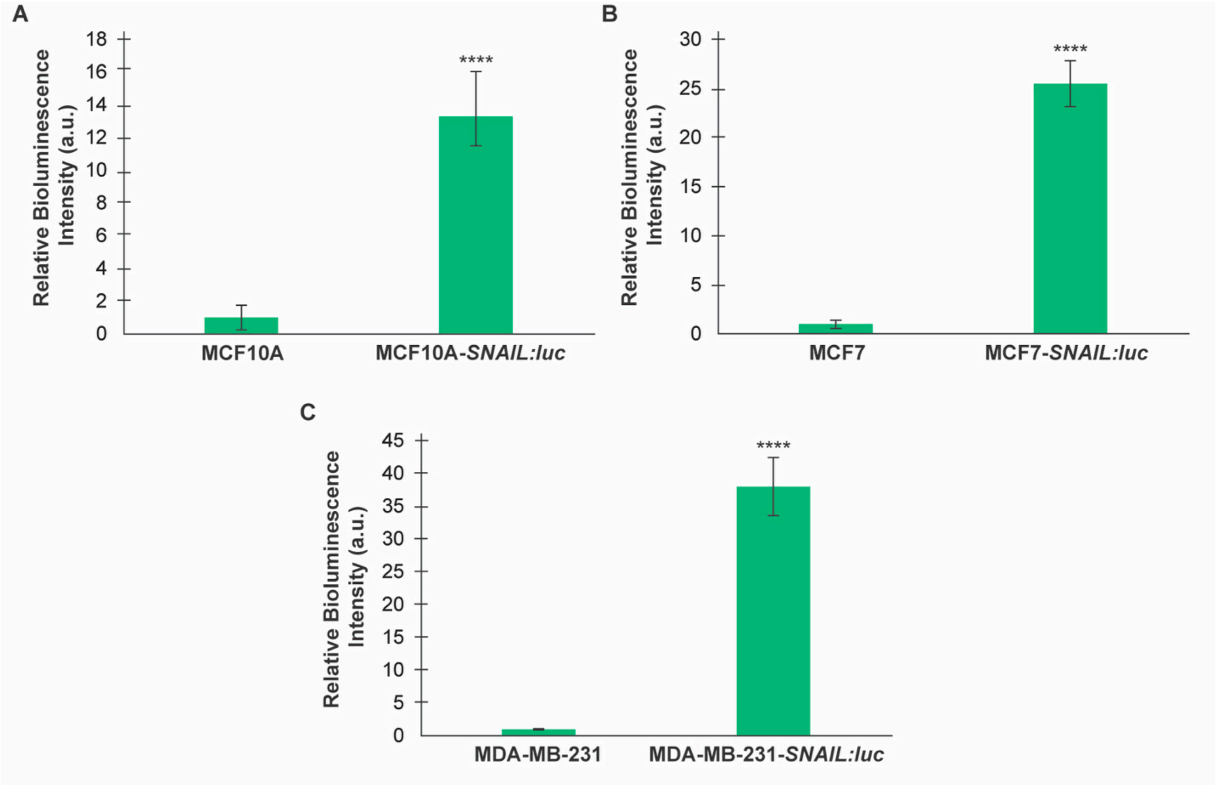

Figure S5. Luciferase assays for MCF10A- (A), MCF7- (B), and MDA-MB-231- (C) *SNAIL:luc* and non-transfected control cells. Each condition is an average of three biological replicates (N=3) plotted with the standard error of the mean. A t-test was performed to compare the averages of the biological replicates for each cell line (ns indicates "not significant," **** $p < 0.0001$).

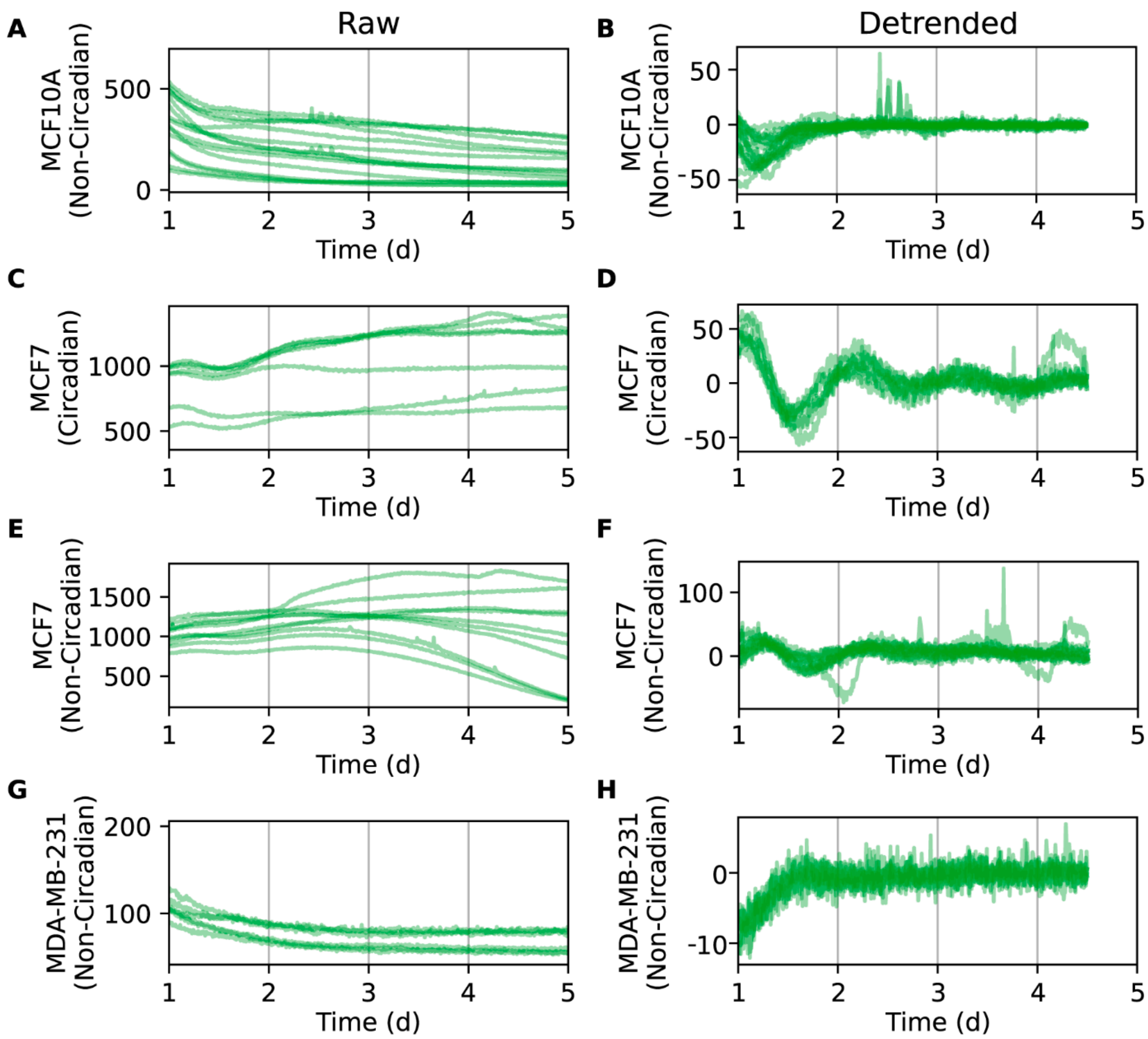

Figure S6. Individual bioluminescence time-series for *SNAIL:luc* in MCF10A (A,B), MCF7 (C-F), and MDA-MB-231 (G,H) cell lines. Shown are raw time-series excluding a 12-h transient (A, C, E, G) and time-series after de-trending by removing the average of a 24-h moving window (B, D, F, H). Seven MCF7 time-series were deemed circadian (C,D; rhythmic with a period in the range of 16 to 32 h). The remaining ten MCF7 (E,F) and all MCF10A (A,B) and MDA-MB-231 (G,H) time-series were non-circadian. (N=15 for MCF10A-*SNAIL:luc*, N=7 for MCF7-*SNAIL:luc* (circadian), N=10 for MCF7-*SNAIL:luc* (non-circadian), N=6 for MDA-MB-231-*SNAIL:luc*)

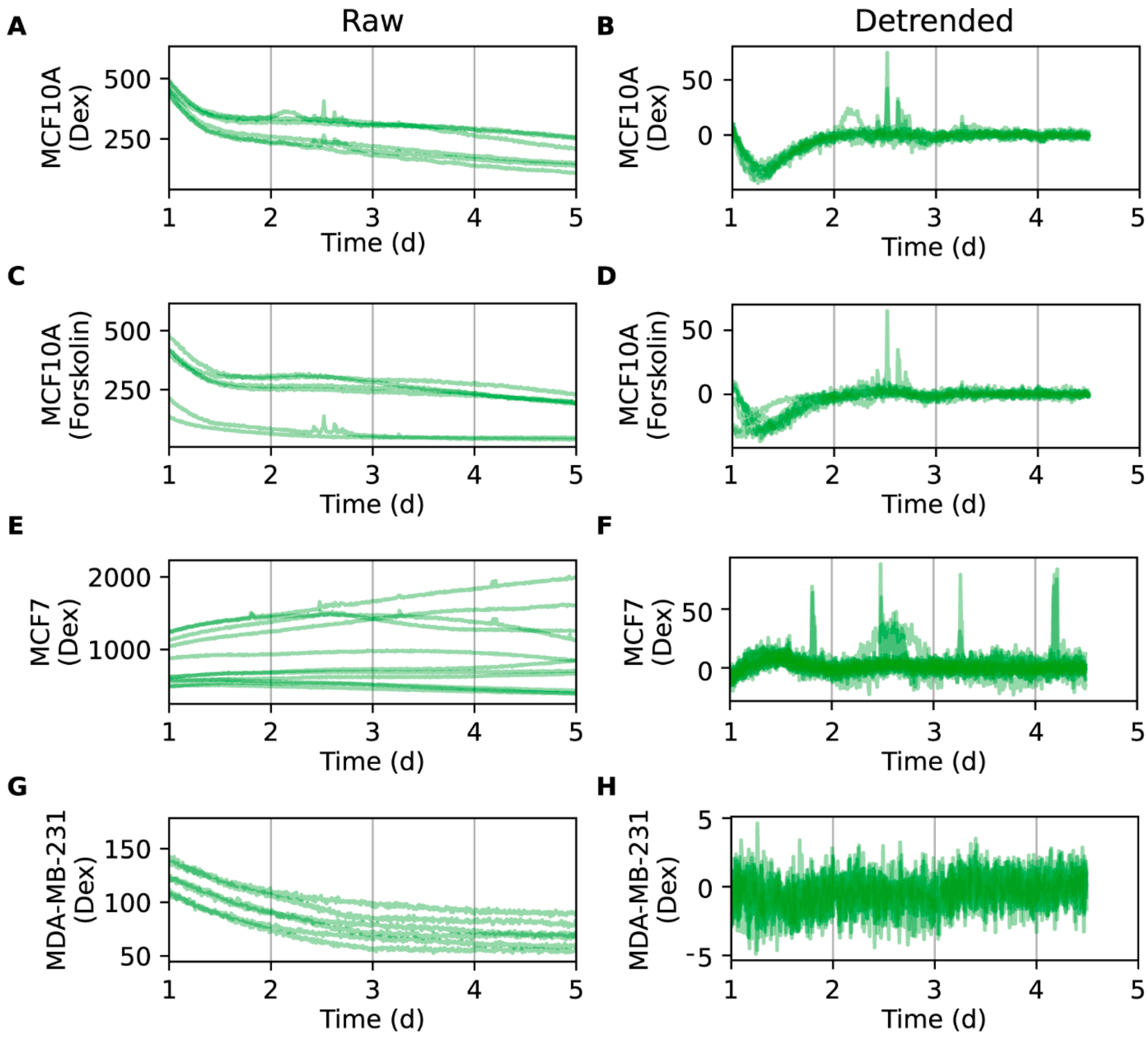

Figure S7. Bioluminescence time-series using alternative synchronization methods for *SNAIL:luc* in MCF10A (A-D), MCF7 (E,F), and MDA-MB-231 (G,H) cell lines. Shown are raw time-series excluding a 12-h transient (A, C, E, G) and time-series after de-trending by removing the average of a 24-h moving window (B, D, F, H). Dexamethasone (Dex) is used for all three lines (A,B,E,F,G,H) and forskolin for MCF10A (C,D). (N=6 for MCF10A-*SNAIL:luc* (dex), N=6 for MCF10A-*SNAIL:luc* (forskolin), N=11 for MCF7-*SNAIL:luc* (dex), N=6 for MDA-MB-231-*SNAIL:luc* (dex)).
